# Gene-Level Regulation of Acupuncture Therapy in Spontaneously Hypertensive Rats: A Whole Transcriptome Analysis

**DOI:** 10.1155/2019/9541079

**Published:** 2019-02-18

**Authors:** Si-Ming Ma, Jing-Wen Yang, Jian-Feng Tu, Na-Na Yang, Yu-Zheng Du, Xue-Rui Wang, Lu Wang, Jin Huang, Cun-Zhi Liu

**Affiliations:** ^1^School of Acupuncture-Moxibustion and Tuina, Beijing University of Chinese Medicine, Beijing, China; ^2^Beijing Hospital of Traditional Chinese Medicine affiliated to Capital Medical University, Beijing, China; ^3^Acupuncture and Moxibustion Department, First Teaching Hospital of Tianjin University of Traditional Chinese Medicine, Tianjin, China

## Abstract

Hypertension is a global health problem. It has been reported that acupuncture at Taichong acupoints (LR3) decreases high blood pressure in spontaneously hypertensive rats. A transcriptome analysis can profile gene expression and its relationship with acupuncture. In this study, rats were treated with 2 weeks of acupuncture followed by regular recording of blood pressure (BP). The mRNA changes in the rostral ventrolateral medulla (RVLM) were evaluated to uncover the genetic mechanisms of acupuncture by using a whole transcript array (Affymetrix Rat Gene 1.0 ST array). BP measurements showed that acupuncture significantly decreased systolic blood pressure (SBP), mean arterial pressure (MAP), and heart rate (HR). In the bioinformatics results, 2371 differentially expressed genes (DEGs) were identified, where 83 DEGs were overlapped among Wistar-Kyoto rats (WKYs), spontaneously hypertensive rats (SHRs), and SHRs + acupuncture rats (SHRs+Acu). Gene ontology (GO) and pathway analysis revealed that 279 GO terms and 20 pathways with significant differences were related to oxidative stress, inflammation, and vascular endothelial function. In addition, coexpressed DEGs networks indicated that Cd4 and Il-33 might mediate the cascade of inflammation and oxidative stress responses, which could serve as a potential target of acupuncture treatment. In conclusion, our study demonstrated that acupuncture is a promising therapy for treating hypertension and could regulate multiple biological processes mainly involving oxidative stress, inflammation, and vascular endothelial function.

## 1. Introduction

Hypertension is a substantial public health problem, affecting over 1 billion people worldwide [1], and results in cardiovascular diseases and chronic kidney disease [2]. As a major independent risk factor for myocardial infarction, stroke, and end-stage renal disease, hypertension is a multifactor disorder that can be triggered by mechanisms in the central nervous systems (CNS) [3]. Neurogenic factors that induce hypertension might be a consequence of abnormal function of the autonomic nervous system, associated with stress, the renin–angiotensin system, insulin resistance, salt sensitivity, and genetic factors [4].

Among all brain regions, the rostral ventrolateral medulla (RVLM) is a critical node of blood pressure (BP) control [5]. Previous studies observed BP changes when RVLM neurons are bulk activated or inhibited with amino acids or GABA-mimetic compounds, while firing RVLM neurons with appropriate optogenetic or pharmacogenetic actuators could also produce the effects of BP fluctuation [5, 6]. Although other regions of the CNS contribute to the regulation of sympathetic tone, a series of experiments clearly showed that the RVLM is the main source of sympathetic activation that plays an irreplaceable role in inducing hypertension [7]. BP changes resulting primarily from variations in sympathetic nerve activity are presumably mediated by monosynaptic projections from the RVLM to preganglionic sympathetic neurons [8]. Abnormalities in the function and structure of the RVLM are closely related to the pathophysiological developing of hypertension [3].

Acupuncture was suggested to elicit integrative effects and modulate neural homeostasis in neurological disease, involving multiple neuroendocrine mechanisms [9, 10]. Previous clinical trials have indicated that acupuncture can effectively lower the systolic blood pressure (SBP) and diastolic blood pressure (DBP) in hypertensive patients [11, 12]. The mechanisms underlying antihypertensive effects of acupuncture involve apoptosis [13], endothelial dysfunction [14], apelinergic system [15], and the opioid system [16, 17]. Our team has recently demonstrated that high BP and oxidative stress in the RVLM of spontaneously hypertensive rats (SHRs) were decreased by acupuncture [18].

To validate the above theories, a bioinformatics study is needed with “big data” about genomics and its relationship with acupuncture, as well as downstream signaling pathways. Mapping and quantifying the transcriptome by microarray provide a digital measurement of the presence and prevalence of transcript [19]. In the RVLM, acupuncture's effect on neurotransmitters has been profiled by a miRNA transcriptome study [20]. Given the inherent pleiotropic effects on gene expression of acupuncture, in the current study, a mRNA analysis was performed to uncover the potential genetic targets of acupuncture in antihypertensive effects.

## 2. Materials and Methods

### 2.1. Animals

All SHRs and Wistar-Kyoto rats (WKYs) were male adults purchased from Vital River Laboratory Animal Technology Co. Ltd (Beijing China). The animals were housed in 12 h light and 12 h dark cycles with a controlled temperature (24±0.5°C), with free access to food and tap water. All animal care and experiments conformed to the US National Institutes of Health (NIH) Guide for the Care and Use of Laboratory Animals and were approved by the Institutional Animal Care and Use Committee of the Capital Medical University.

### 2.2. Acupuncture Treatment

30 rats (12 weeks) were weighed and block-randomized with computer software (Graphpad Software, Inc., La Jolla, CA, USA) into 3 groups: Wistar-Kyoto rats (WKYs), spontaneously hypertensive rats (SHRs), and SHRs treated with acupuncture (SHRs+Acu). To eliminate the effect of constraint stress, an acupuncturist handled and fondled animals (1-2 hours/day, 2-3 days) before the acupuncture treatment. After the adaptive training, a 2-week period of acupuncture therapy was administrated to conscious rats at 3 p.m. to 4 p.m. each day. Before treatment, the bodies of rats were immobilized softly by an assistant and scrubbed with a 75% alcohol disinfectant at the acupoints. Then acupuncture was performed at bilateral LR3s with an intradermal needle by the acupuncturist. LR3 is located between the first and the second metatarsal bone of dorsal foot. The needles were inserted to a 5 mm depth and retained for 20 min before removal.

### 2.3. BP and Heart Rate Measurements

To investigate the effects of acupuncture on BP and sympathetic activity, a floating polyethylene catheter was inserted into the lower abdominal aorta, and the transmitter was implanted into the abdominal cavity. Seven days after the implantation, systolic blood pressure (SBP), mean arterial pressure (MAP), and heart rate (HR) were recorded for 2 h from 8 a.m. to 10 p.m. by a radio telemetry system (Data Sciences International, Saint Paul, Minnesota, USA) every 2 days. For BP and HR analysis, the system measured the mean values of these parameters for 2 hours in each measurement. The experimental staffs were blinded to group assignment.

### 2.4. Total RNA Extraction and mRNA Microarray

At the 14th day of acupuncture treatment, rats were euthanized with a sodium pentobarbital overdose (100mg/kg). Rat brains were removed and placed on dry ice. According to atlas, one 1 mm^2^ region was obtained in the RVLM (bregma -12 to -12.96mm) from a 1 mm-thick slice at the level of brainstem via micropunch technique [21]. Total RNAs from RVLM were extracted using RNeasy Mini kit (Qiagen, Valencia, CA, USA). The amount and the integrity of total RNA were quantified and evaluated using a spectrophotometer (Beckman Coulter, Fullerton, CA, USA) and an Agilent 2100 bioanalyzer (Agilent Technologies, Santa Clara, CA, USA), respectively.

The total RNA was extracted using a TRIzol reagent (Invitrogen, Carlsbad, Canada). Hybridization and scanning of the chips were performed by Affymetrix Rat Gene 1.0 ST Array, which contained 59,302 gene-level probe sets.

### 2.5. Analysis of Differentially Expressed mRNA

To discern the genes that are differentially expressed, we chose p-value < 0.05 by ANOVA as well as the fold change <1.2 as the threshold screening among the 3 groups. The false discovery rate (FDR) was also computed in view that the smaller the FDR, the smaller the error in assessing the p-value.

### 2.6. Analysis of Series Test of Cluster (STC) of DEGs

Based on RVM (random variance model) corrective ANOVA, significant differentially expressed genes (DEGs) among the 3 groups were selected. According to the signal density tendency of genes in these differently treated groups, expression values were converted into log⁡2 ratio, and 16 unique model profiles were identified based on the actual number of genes assigned to each model. Fisher's exact test and multiple comparison tests were performed to define the significant profiles.

### 2.7. qRT-PCR Analysis

In qRT-PCR analysis of validation, the total mRNA (2 *μ*g) of samples was conducted to a reaction with AMV reverse transcriptase (Promega, Madison, WI) for first-strand cDNA synthesis. For each reaction, cDNA of sample was added to 2x SYBR master mix (Takara, Otsu, Shiga, Japan) and analyzed by using a BIORAD iCycler iQ5 (Bio-Rad, Hercules, CA). The sequences accessions of the primers are Cox5b (5′-GGAGGTGGTGTCCCTACTGA-3′ forward, 5′-GGAGGTGGTGTCCCTACTGA-3′ reverse), Sirt6 (5′-GCCGTCTGGTCATTGTCA-3′ forward; 5′-GCCGTCTGGTCATTGTCA-3′ reverse), Nf1 (5′-TTCGATACACTTGCGGAAAC-3′ forward; 5′-CACATTGGCAAGAGCCATAG-3′ reverse), Gabbr1 (5′-GGCTTTAGTCTGGGCTATG-3′ forward; 5′-GGCTTTAGTCTGGGCTATG-3′ reverse). Gene levels were normalized to that of *β*-actin. All samples were run in duplicate.

### 2.8. Gene Ontology (GO) Analysis and Pathway Analysis

Gene ontology (GO, www.geneontology.org/) analysis and Kyoto Encyclopedia of Genes and Genomes (KEGG, http://www.genome.jp/kegg/) pathway analysis were performed to identify the functions and associated enriched pathways of focused mRNAs. The z-score of threshold for mRNA expression was defined as ±2.0.

### 2.9. The Topological Analysis of Coexpressed DEGs

Gene coexpression networks were built to identify the interactions among 83 DEGs. We calculate the Pearson correlation and choose the significant correlation pairs to construct the networks. Degree centrality is defined as the link numbers that one gene has to the other. Besides, k-cores of networks were calculated to show the simplified topological characteristics.

### 2.10. Data and Statistical Analysis

Statistics in microarray and bioinformatic analyses were performed by 1-way analysis of variance (ANOVA) using Affymetrix® Expression Console™ TAC (Affymetrix® Expression Console™), followed by the least significant difference (LSD) test. The BP and HR data were analyzed by 2-way ANOVA with repeated measures, and followed by the Scheffe multiple-range test for post hoc assessment of individual means. Significant differences were considered at p < 0.05.

## 3. Results

### 3.1. The Effects of Acupuncture on BP and HR in Rats

**Figure 1:**
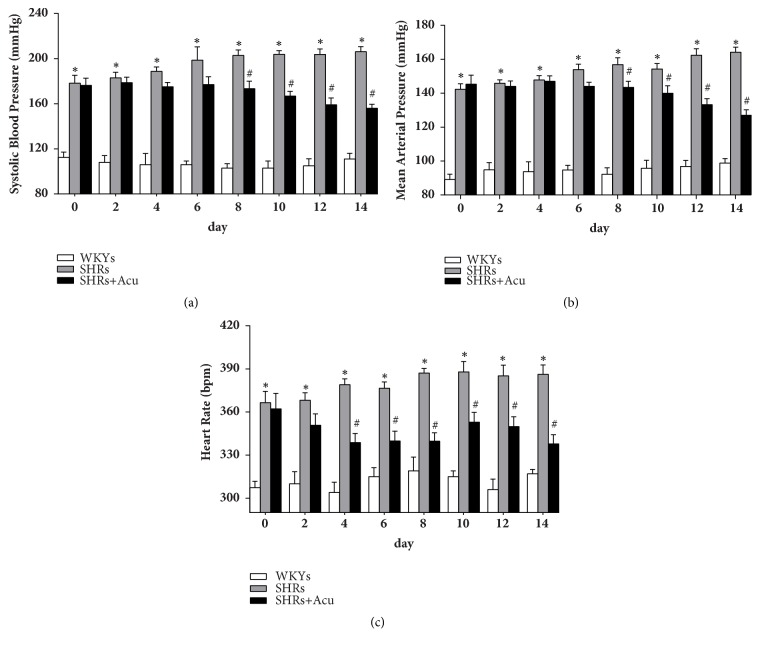
The antihypertensive effects of acupuncture on spontaneously hypertensive rats (SHRs). (a) Systolic blood pressure; (b) mean arterial pressure; (c) heart rate. N=10, data are represented as mean ± SEM, *∗p* < 0.05 SHRs vs WKYs, #*p* < 0.05 SHRs+Acu vs SHRs (Scheffe multiple-range analysis), respectively.

To assess the antihypertensive effect of acupuncture in hypertension, radiotelemetry recording was performed before and during the experiment. As shown in Figure 1, acupuncture at LR3 in SHRs significantly decreased SBP and MAP from day 8 to the end of the treatment compared with SHRs (Figures 1(a) and 1(b)). HR also underwent a persistent decrease from day 4 in SHRs+Acu group (Figure 1(c)). But the normal BP and HR of WKYs were not altered by acupuncture.

### 3.2. DEGs Screening among 3 Groups

**Figure 2:**
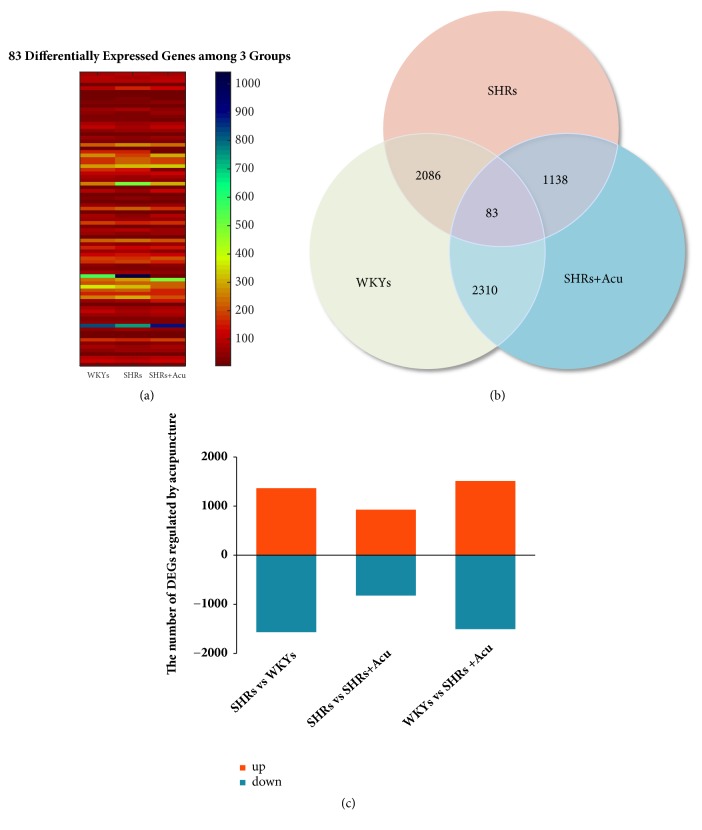
The genes expression that might be regulated by acupuncture in 3 conditions was profiled. (a) 83 DEGs among 3 groups (ANOVA analysis, fold change < 1.2, *p* < 0.05); (b) the number of overlapped DEGs between each pair of groups; (c) up/downregulated DEGs in each pair of compared groups.

In the microarray analysis, genes expression that might be regulated by acupuncture treatment in the 3 groups was profiled. A threshold was defined as FD⩽0.001 and log⁡2 Ratio*⩾*1. As a result, a total of 2371 genes were acquired in all 3 groups (p < 0.05), the top 10 DEGs are Haghl, Fmo2, Mgst3, Dusp12, Cdkn1a, Glo1, Crot, Nmnat1, Eapp, Mettl7a (Table 1). To identify genes that were differentially regulated under the conditions of hypertension and acupuncture, a crossover comparison was performed in the following 3-paired groups: WKYs and SHRs, SHRs and SHRs+Acu, WKYs and SHRs+Acu. There was a number of 2169 (SHRs vs WKYs), 1221 (SHRs vs SHRs+Acu), and 2393 (WKYs vs SHRs+Acu) DEGs, as the Venn diagram demonstrates that they are coexpressed, respectively, in each pair of groups (Figure 2(b)), of which 83 DEGs were overlapped among 3 groups (Figure 2(a)). The number of up/downregulated DEGs was also calculated in each group (Figure 2(c)).

### 3.3. DEGs Clusters of Differentially Expression Patterns

**Figure 3:**
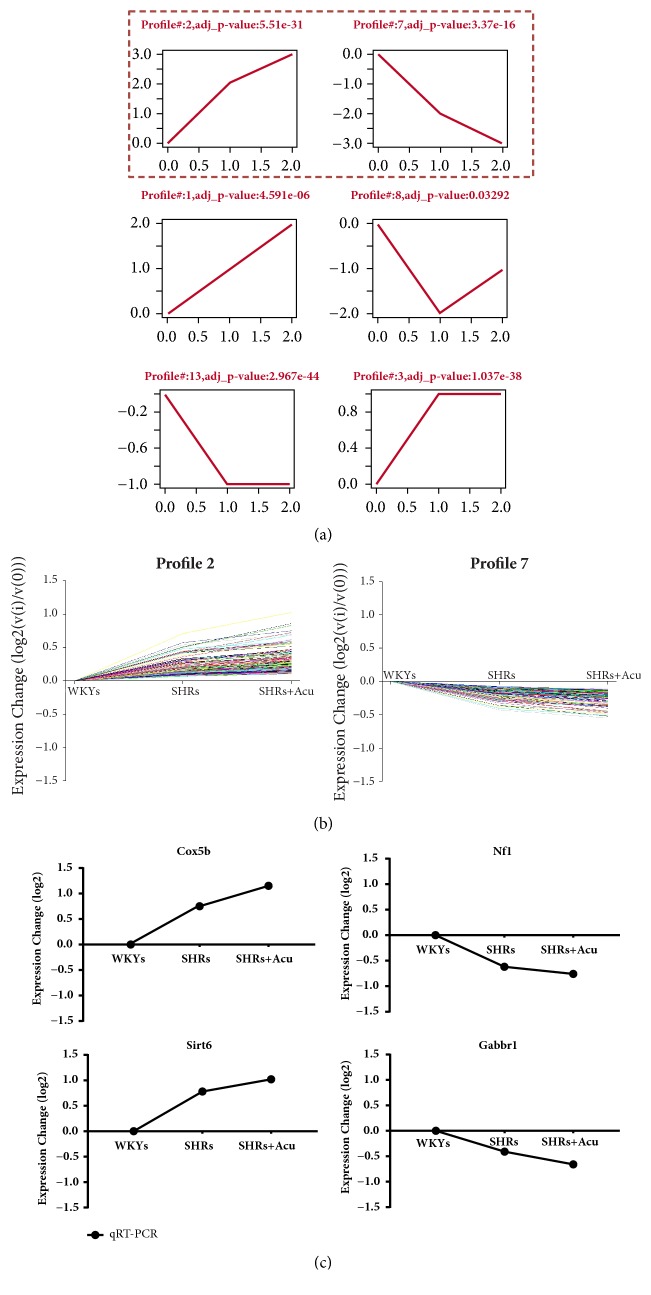
Significant profiles and genes that could be regulated by acupuncture. (a) The ascending trend of profile 2 is mirrored by the descending trend of profile 7; (b) 226 genes were classified into profile 2, and 197 genes were classified into profile 7; (c) validation of 4 representative genes by qRT-PCR, including Cox5b and Sirt6 in profile 2, Nf1 and Gabbr1 in profile 7.

To profile the DEGs and choose significant gene clusters with parallel expression patterns induced by acupuncture in SHR rats, 16 profiles with temporal expression patterns were defined in line with the trend of expression changes among the 3 groups. Temporal expression patterns and the significance of the genes were obtained by STEM software. The series test of cluster showed that 6 significant clusters were considered as potential profiles that could be affected by acupuncture (p < 0.05, Figure 3(a)). Notably, 226 genes were classified into the profile 2 and were gradually upregulated from WKYs to SHRs and SHRs+Acu. Meanwhile, profile 7 which included 197 genes was consistently decreased from the WKYs to SHRs and SHRs+Acu (Figure 3(b)).

### 3.4. Validation of Clusters Profiling Results by qRT-PCR

To verify the microarray profiling results, four significantly expressed genes that concern cardiovascular disease, Cox5b and Sirt6 in profile 2, Nf1 and Gabbr1 in profile 7, were evaluated by qRT-PCR after gene cluster analysis. The quantitative results of each expressed mRNA were consistent with the variation of its original clusters (Figure 3(c)).

### 3.5. GO and Pathway Analysis

**Figure 4:**
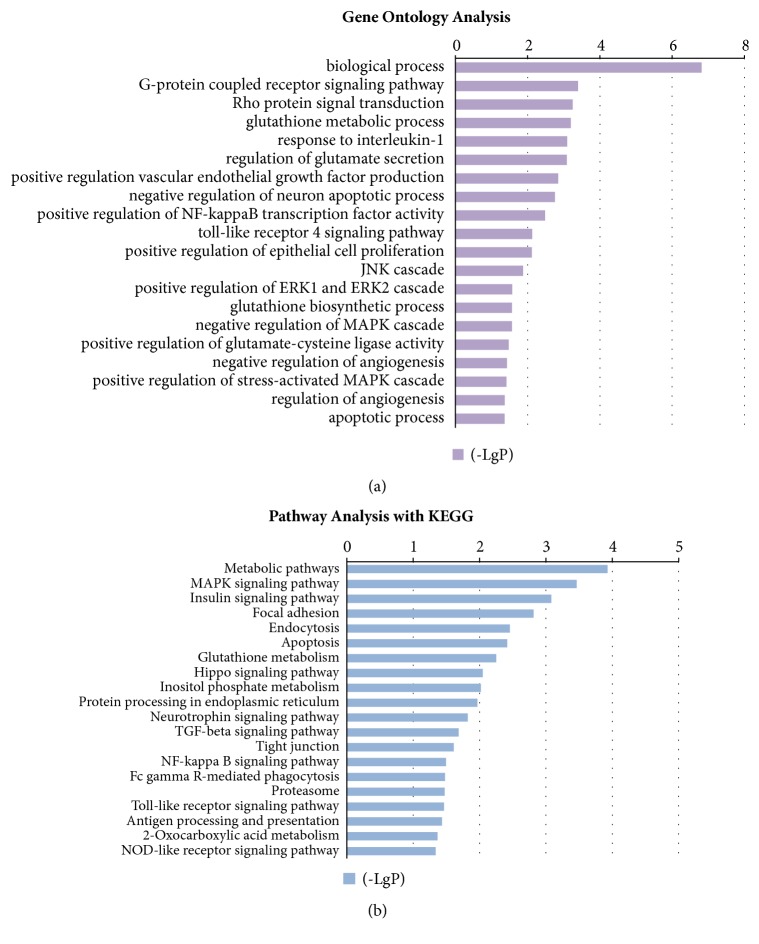
Bioinformatics analysis of gene ontology (GO) analysis and pathway analysis with KEGG. (a) GO analysis reflects functional characteristics of 20 representative overrepresented genes that might be regulated by acupuncture; (b) pathway analysis demonstrates 20 significantly signaling pathways that acupuncture might be involved in.

To examine the characteristics of DEGs, functional classification of intersecting genes was performed using the gene ontology (GO) tool. A total of 1640 GO terms were identified, among which 279 GO terms were significant (p<0.05). In Figure 4(a), we show the 20 significantly overrepresented Go in terms of biological function. Top 10 GO terms are biological process, G-protein receptor signal transduction, Rho protein signal transduction, glutathione metabolic process, response to interleukin-1, regulation of glutamate secretion, positive regulation of vascular endothelial growth factor production, negative regulation of neuron apoptotic process, positive regulation of nuclear factor-kappaB (NF-kappaB) transcription factor, and toll-like receptor 4 (TLR4) signaling pathway. Accordingly, several representative genes (Fgfr2, Ptgsl, Ripk2, Vegfa, Nf1, Gabbr1, Gclm, Grm1) within specific GO terms are listed in Table 2.

To further understand the biological functions of the differentially expressed genes, we performed a KEGG pathway enrichment analysis on the basis of the intersection of genes (Figure 4(b)). A total of 177 pathways were identified, among which 20 pathways were significant (p<0.05). The top 10 significantly signaling pathways were, respectively, associated with metabolic pathways, a mitogen-activated protein kinases (MAPK) signaling pathway, insulin signaling pathway, focal adhesion, endocytosis, apoptosis, glutathione metabolism, Hippo signaling pathway, and inositol phosphate metabolism, and protein processing in endoplasmic reticulum was identified.

### 3.6. The Topological Analysis of Coexpressed DEGs

**Figure 5:**
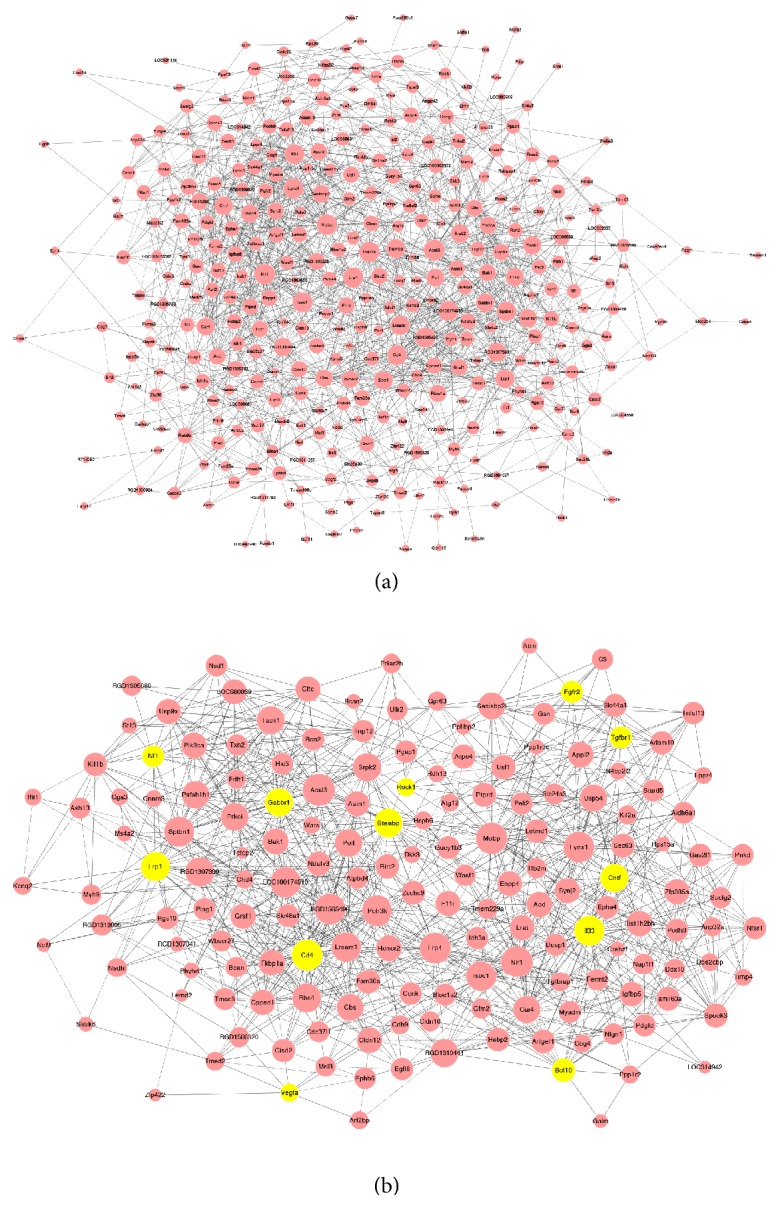
(a) The coexpressing gene network of 347 DEGs in hypertension; (b) the subnetwork of coexpressing DEGs that are at the top of list. Circles represent genes (pink circles: DEGs, yellow circles: DEGs on the top of list), the edges between nodes represent gene-gene interaction, and the diameter of circles represents the degree of gene-gene interaction.

The dynamic real network was built to manifest the interaction of DEGs according to the data of a GO analysis (absolute value of interaction*⩾*0.800) (Figure 5(a)). We found that Cd4 and Il3 were connected with 39 genes (Degree =39) and occupied the leading position of modulation (k-core =12) in gene networks (Figure 5(b)). Since the expression of these 2 genes has been widely suggested to be of great importance to the formation of hypertension, we hypothesized that they may be involved in the antihypertensive effect of acupuncture.

## 4. Discussion

Acupoints selecting is essential for acquiring therapeutic effect in acupuncture therapy [22]. LR3 proved to be effective for treating hypertension. In stress-induced prehypertension model, needling at LR3 and Quchi (LI11) could reduce BP by targeting the gene expression of heart and hypothalamus [23, 24]. Consistent with previous studies, we selected LR3 and found that acupuncture significantly decreased the SBP, MBP, and HR in SHRs compared with WKYs, without any impact on normal BP. Moreover, some imaging studies showed that the effects of acupuncture on BP might involve altered cerebral or urinary metabolism [25, 26]. The molecular mechanism may be due to an increase in antioxidant enzyme in medulla according to a proteomic study [27]. Genetic information from* in situ* study was yielded by the microarray approach that was performed in the RVLM, and demonstrated that only a minority out of the 2371 DEGs were characterized as being associated with hypertension. Our analysis highlights once again that specific genes and biological processes may constitute the therapeutic effects of acupuncture on development of neurogenic hypertension. Of note, the bioinformatics results implicated that a large number of pathways and GO terms are associated with oxidative stress and inflammation. For instance, MAPK signaling pathway, JNK or ERK cascade, and glutathione metabolism are acknowledged as the fundamental processes mediating oxidative stress responses, while NF-kappa B and Toll-like receptor signaling pathways, and interleukin-1 could be the direct mediator of cellular inflammation or promotor of oxidative stress [28]. Notwithstanding the disperse evidence, these results provide a framework for further validation.

### 4.1. The BP Regulation of Acupuncture Might Be Involved in Multiple Gene Clusters

Cluster analysis was used to find out the transcriptional evidence of acupuncture's effect on BP. Among 6 significant profiles, interestingly, the descending of profile 7 is mirrored by the ascending tendency of profile 2. We hypothesized that the phenomenon of their negative correlation might reflect the functional relation between 2 profiles in signaling pathway of hypertension.

To further answer the questions, 4 representative genes were selected (Cox5b, Sirt6, Nf1, and Gabbr1) to perform qRT-PCR due to their high correlation with oxidative stress or hypertension. Cytochrome c oxidase 5b (Cox5b) abnormal activity in hypertrophic hearts has been observed in SHRs [29]. Sirt6, predominantly expressed in nucleus, inhibits inflammation via reactive oxygen species (ROS) and Akt signaling pathway [30]. It is well known that Nf1 mutations lead to a common autosomal dominant disorder, neurofibromatosis type 1. The neurofibromatosis type 1 patients have an increased incidence of cardiovascular diseases, including obstructive vascular disorders and hypertension [31]. The gamma-aminobutyric acid B-type receptor unit 2 (Gabbr1) expressed in paraventricular nucleus (PVN) is important in controlling sympathetic activity, and its upregulation attenuated sympathoexcitation in chronic heart failure [32, 33]. Based on a series of elegant studies, such selections are relevant and sufficient for PCR validation. As expected, the expression scenario of these mRNA in 2 profiles is consistent with that in cluster analysis. This congruency suggests that the balance of some genetic subgroups, but not single gene, participates in maintaining blood pressure and could be regulated by acupuncture at LR 3.

GO analysis exhibited some redox-related genes that might be regulated by acupuncture. For example, Ripk2, also known as Rip2, is a kinase participating in inflammatory response signaling. Knocking down Ripk2 could reduce phosphorylation of p38 MAPK, ERK, and IkappaB Alpha and result in a decrease of IL-12 [34]. Moreover, suppression of endogenous Ripk2 significantly decreased apoptosis [35]. Fgfr2, a gene for encoding fibroblast growth factor receptor 2 (FGFR2), proved to regulate several growth-related signaling pathways in cancer [36]. The identification of specific mRNA sequence of Fgfr2 activates oncogenes through MAPK and PI3K/mTOR pathways in neuronal glial tumors [37]. Kobayashi T et al. demonstrated that Gclm knockout mice have long-term depletion of myocardial glutathione levels, which exacerbates myocardial oxidative stress [38] and dysfunction of the pressure-overloaded heart [39].

### 4.2. Oxidative Stress and Inflammation: The Key Combating in Antihypertensive Effect of Acupuncture

One main purpose of this study was to summarize the common and differential genetic factors that could be modulated by acupuncture through refining a huge dataset. Oxidative stress is an important contributor to hypertension, diabetes, and aging [40]. The ROS generated by NADPH oxidases (NOX) could mediate redox-sensitive signaling pathways through activating angiotensin II and cause endothelial dysfunction and vascular inflammation [41, 42]. This imbalance would be deleterious when inflammation is sustained. Although several signaling pathways affect the antihypertensive effects of treatment, the cross-talk between oxidative stress and inflammation might be a central mechanism of hypertension modulation for acupuncture.

Among 20 screened signaling pathways, MAPK signaling pathway is at the top of the KEGG list. As a critical inducer of oxidative stress, the activation of MAPK in RVLM leads to tissue and organ impairment in the form of phosphorylation [43]. Recent work of our group further revealed that acupuncture elicited antihypertensive effects and alleviated oxidative stress, especially via p38 MAPK and Erk1/2 signaling pathway in RVLM [18]. In addition, with the high response to oxidative stress, NF-KappaB signaling pathway is implicated in a series of metabolic syndromes. It has been well established that chronic inhibition of NF-KappaB in PVN attenuated sympathoexcitation by downregulating NOX and proinflammatory cytokine in hypertension [44]. This epigenetic changes induced by acupuncture now have been associated with MicroRNA-339 and Sirt2 (upstream of NF*κ*B) [45].

Furthermore, 2 redox-sensitive pathways screened by microarray, including glutathione metabolism and the neurotrophin pathway, contribute to oxidative stress in hypertension. Glutathione is a major intracellular thiol-disulfide redox cofactor for many antioxidant enzymes and is considered to be a key node in combating increased blood pressure, not only for its capacity of free radical scavenging, but also by controlling nitric oxide (NO) bioavailability [46]. A proteomic study in hypertension indicates that glutathione S-transferase M5 could be upregulated by acupuncture [47]. Neurotrophin factors, such as brain-derived neurotrophin factor (BDNF), are acknowledged to be a sensitive marker of oxidative stress. They increase, prior to the BP rise, and enhance the survival of noradrenergic neurons hyperinnervated by sympathetic nerves [48, 49]. A miRNA study profiled that acupuncture could restore high BP through activating neurotrophin signaling pathway [20]. Therefore, our study reinforced the idea that acupuncture affects hypertension by regulating signaling pathways concerning oxidative stress.

### 4.3. Vascular Endothelial Dysfunction: Initiated by Oxidative Stress

Endothelial dysfunction induces the deterioration of endothelium-dependent vasodilation including the aberrant metabolism of nitric oxide and imbalance of vasomotor factors [50]. For example, as an angiogenic factor, vascular endothelial growth factor-A (VEGFA) regulates BP by promoting vasodilation. The increased value of VEGFA, accompanied by decreased NO, could be observed in hypertension. Ptgs1 is engaged in regulation of BP by encoding cyclooxygenase-1 (COX-1) [51, 52]. The COX-derived prostanoids further mediate vascular tone in programmed hypertension [19]. These emerging studies implicated the importance of endothelial dysfunction in hypertension, where the oxidative stress serves as the main factor of pathogenesis [28]. It has been evidenced that the overproduction of ROS is correlated with the dysfunctional endothelial NOS (eNOS) and results in the reduction of vascular relaxation [53]. During the course of pathological changes, acupuncture could attenuate BP by protecting endothelial function from oxidative stress in SHRs [14]. In accordance, GO annotation showed the beneficial effects of acupuncture in vascular endothelium and, additionally, implied that a tiny stimulation of acupuncture could cause multiple genes cascade due to the activation of oxidative stress in hypertension.

### 4.4. Unproved but Promising Pathways: All Roads Lead to Antioxidative Effect of Acupuncture

Our study also reported several redox-related signaling pathways that have not been mentioned in prior studies of the mechanisms of acupuncture for treating hypertension. First, increased BP is commonly accompanied by risk factors such as insulin resistance or dyslipidemia especially in SHRs and essential hypertension in human [54]. The characteristics of oxidative stress and the impaired PI3 pathway may favor the insulin resistance in rats and patients with hypertension [55, 56]. Second, the main function of the Hippo signaling pathway has been expanded to modulating cell proliferation, differentiation, and migration in many organs [57]. Its function of negative regulation of cardiomyocytes survival was proposed to be associated with inhibiting Yes-associated protein (YAP)-FoxO1 interaction and antioxidant gene expression [58]. Third, NOX4 upregulation would induce the unfolded protein response (UPR), causing endoplasmic reticulum (ER) stress in vascular smooth muscle cells, which was mentioned frequently in the last few years for its role in BP elevation and vascular injury in SHR [59]. Fourth, TGF-beta signaling pathway also activates NOX-induced oxidative stress and accelerates endothelial dysfunction in hypertension [60–62]. Thereby, these putative pathways could be some new targets for acupuncture treatment, and their function in acupuncture effectiveness and oxidative stress should be clarified by enough experiments.

### 4.5. Homeostasis: The Macroscopic Mechanism of Acupuncture Treatment

The topological analysis illustrated an unanticipated outcome; that is, Cd4 and Il33 take control of the gene network. Previous investigations indicated that the immune system mediates human essential hypertension via the activation of innate and adaptive immunity in periphery [63]. As a main T-regulatory lymphocytes (Treg), CD4^+^ lymphocytes that express choline acetyltransferase (ChAT) induce the vasodilation by relaying the signals from vagus nerve [64]. The gene expression of CD4^+^CD44^hi^CD62L^lo^ T helper cells would determine this distinct type of T-cell [65]. Furthermore,* In vitro* experiment identified a feedback loop, in which IL-33, in addition to its anti-inflammatory and protective effects in cardiovascular system, stimulates the expanding of tumorigenicity 2 (ST2)-expressing Treg during interactions with CD4^+^ [66].

In the CNS, however, the tight conjunction of blood brain barrier (BBB) makes it difficult for T-cells or IL-33 to penetrate into the brain parenchyma. A widely accepted hypothesis is that circumventricular organs (CVOs) such as the subfornical organ (SFO), which lack the normal BBB, are apt to be triggered by Ang II and induce the change of BBB filterability in other brain regions [67]. Indeed, T-lymphocyte infiltration was found in SFO tissue [68], and SFO-PVN-RVLM pathway induced by Ang II-ROS has been described in a few researches in neurogenic hypertension [67, 69]. This delineation of integrity that combines immunity, inflammation, and oxidative stress is consistent with the homeostasis theory in acupuncture treatment undoubtedly. We are cognizant that the expression of these genes might provide some small pieces of puzzle for mechanism of blood pressure maintaining and for mechanism of acupuncture therapeutic effects too.

## 5. Conclusion

A comprehensive bioinformatic analysis revealed that genes expression related to oxidative stress, inflammation, and vascular endothelial function and downstream signaling pathways it is involved in might be associated with the antihypertensive effects of acupuncture. Our present study enriched the understanding of the antihypertensive mechanisms of acupuncture. More studies are needed to address the relationship between these genes and hypertension, as well as the beneficial role of acupuncture in gene expression and pathway regulation.

## Figures and Tables

**Table 1:** The differentially expressed genes among the 3 groups.

Gene Symbol	Gene Description	p-value	FDR	WKYs	SHRs	SHRs+Acu
Haghl	hydroxyacylglutathione hydrolase-like	1.00E-07	4.08E-05	35.99	22.81	23.69
Fmo2	flavin containing monooxygenase 2	1.00E-07	4.08E-05	9.86	7.32	6.74
Mgst3	microsomal glutathione S-transferase 3	1.00E-07	4.08E-05	377.27	527.33	567.29
Dusp12	dual specificity phosphatase 12	1.00E-07	4.08E-05	76.85	104.65	114.5
Cdkn1a	cyclin-dependent kinase inhibitor 1A	1.00E-07	4.08E-05	21.74	33.7	31.53
Glo1	glyoxalase 1	1.00E-07	4.08E-05	229.94	165.71	177.26
Crot	carnitine O-octanoyltransferase	1.00E-07	4.08E-05	58.96	34.31	38.22
Nmnat1	nicotinamide nucleotide adenylyltransferase 1	1.00E-07	4.08E-05	103.73	171.11	173.61
Eapp	E2F-associated phosphoprotein	1.00E-07	4.08E-05	292.06	199.04	208.1
Mettl7a	methyltransferase like 7A	1.00E-07	4.08E-05	52.61	79.12	75.12

**Table 2:** Partial genes and pathways screened by GO and pathway analysis.

Gene name	GO Term	Pathway Name
Fgfr2	epithelial cell differentiation	MAPK signaling pathway
	positive regulation of epithelial cell proliferation	
	response to stress	
	protein phosphorylation	
	JNK cascade	
	positive regulation of ERK1 and ERK2 cascade	
	negative regulation of MAPK cascade	
	regulation of Ras GTPase activity	
	positive regulation of stress-activated MAPK cascade	
Vegfa	positive regulation of I-kappaB kinase/NF-kappaB cascade	NF-kappaB signaling pathway
	regulation of apoptotic process	
	immunoglobulin mediated immune response	
	positive regulation of T cell activation	
	protein autophosphorylation	
	apoptotic process	
	apoptotic process	
Nf1	glutathione metabolic process	Glutathione metabolism
	negative regulation of neuron apoptotic process	
	response to oxidative stress	
	glutathione biosynthetic process	
	regulation of blood vessel size	
	positive regulation of glutamate-cysteine ligase activity	
Ptgsl	positive regulation of transcription from RNA polymerase II promoter	Neurotrophin signaling pathway
	positive regulation of smooth muscle cell proliferation	
Ripk2	response to interleukin-1	Neurotrophin signaling pathway
	toll-like receptor 2 signaling pathway	
	positive regulation of NF-kappaB transcription factor activity	
	toll-like receptor 4 signaling pathway	
	positive regulation of stress-activated MAPK cascade	
Gabbr1	positive regulation of glial cell proliferation	Insulin signaling pathway
	response to interleukin-1	
Gclm	angiogenesis	Insulin signaling pathway
	negative regulation of neuron apoptotic process	
	positive regulation of NF-kappaB transcription factor activity	
	negative regulation of apoptotic process	
	protein phosphorylation	
Grm1	JNK cascade	Insulin signaling pathway
	protein phosphorylation	
	negative regulation of angiogenesis	

## Data Availability

The data used to support the findings of this study are available from the corresponding author upon request.

## Supplementary Materials

Supplementary Materials The supplementary information to support the results of transcriptome analysis is available in the online version of this paper.
